# Neuroendocrine regulations in tissue-specific immunity: From mechanism to applications in tumor

**DOI:** 10.3389/fcell.2022.896147

**Published:** 2022-08-22

**Authors:** Si-Qing Liu, Bei Li, Juan-Juan Li, Si Sun, Sheng-Rong Sun, Qi Wu

**Affiliations:** ^1^ Department of Breast and Thyroid Surgery, Renmin Hospital of Wuhan University, Wuhan, Hubei, China; ^2^ Department of Pathology, Renmin Hospital of Wuhan University, Wuhan, Hubei, China; ^3^ Department of Clinical Laboratory, Renmin Hospital of Wuhan University, Wuhan, Hubei, China; ^4^ Tongji University Cancer Center, Shanghai Tenth People’s Hospital of Tongji University, School of Medicine, Tongji University, Shanghai, China.

**Keywords:** neuroendocrine regulation, neurotransmitter, neuropeptide, tissue-specific immunity, cancer

## Abstract

Immune responses in nonlymphoid tissues play a vital role in the maintenance of homeostasis. Lots of evidence supports that tissue-specific immune cells provide defense against tumor through the localization in different tissue throughout the body, and can be regulated by diverse factors. Accordingly, the distribution of nervous tissue is also tissue-specific which is essential in the growth of corresponding organs, and the occurrence and development of tumor. Although there have been many mature perspectives on the neuroendocrine regulation in tumor microenvironment, the neuroendocrine regulation of tissue-specific immune cells has not yet been summarized. In this review, we focus on how tissue immune responses are influenced by autonomic nervous system, sensory nerves, and various neuroendocrine factors and reversely how tissue-specific immune cells communicate with neuroendocrine system through releasing different factors. Furthermore, we pay attention to the potential mechanisms of neuroendocrine-tissue specific immunity axis involved in tumors. This may provide new insights for the immunotherapy of tumors in the future.

## 1 Introduction

Tumors develop in complicated tissue environments, which they rely on for growth, invasion and metastasis (Quail and Joyce, 2013). The tumor microenvironment (TME) is a heterogeneous ecosystem composed of all the structures at the site and those that are recruited to the area—immune cells, mesenchymal stem cells, vascular vessels, nerves and matrix components (Hanahan and Coussens, 2012). Tumors are derived from the complex interactions that occur between them (Gysler and Drapkin, 2021). With the constant exploration of new ways to treat tumors, therapies targeting the TME have emerged as a promising approach for cancer treatment for the past few years (Bejarano et al., 2021). Research has progressed, but there are remaining unknowns. The composition of the TME is being elucidated, with the neurological, immunological and microbiological components being identified. However, other characteristics and the dynamic evolution of the TME require further in-depth exploration.

Tissue-specific immunity occurs when immune cells establish permanent tissue residency in different organs, thus creating a defensive system (Klose and Artis, 2020). Numerous immune cells reside in nonlymphoid tissues. These tissue-resident populations do not recirculate and adopt a unique phenotype that is distinct from immune cells in the blood or lymphatic system (Mackay and Kallies, 2017). The origin of tissue-resident immune cells is complex and includes tissue innate immune cells and immune cells that have migrated from the peripheral blood. Innate immune cells, including macrophages, dendritic cells (DCs) and innate lymphoid cells (ILCs), exhibit tissue-specific subset compositions in the lung, skin, intestines and comprise the early responders to pathogen encounters. Adaptive immune cells, such as T cells and B cells, also have tissue-resident subsets that help to build immune memory. For example, tissue-resident memory T cells persist as tissue-resident populations in mucosal and exocrine sites, while memory B cells predominate in the intestines. In general, the human immune system is localized in a tissue-specific manner in diverse sites (Weisberg et al., 2021). Functionally, tissue-specific immunity is observed to play a part in tumor development through various regulatory mechanisms (Lei et al., 2020; Liu et al., 2022a).

Recently, the contribution of nerves to the pathogenesis of malignancies has emerged (Zahalka and Frenette, 2020). Several landmark studies have demonstrated that the nervous system plays an active role in tumorigenesis (Reavis et al., 2020). Meanwhile, some studies have suggested that tumor innervation is associated with accelerated tumor progression in multiple cancers (Albo et al., 2011; Magnon et al., 2013; Huang et al., 2014; Kappos et al., 2018; Renz et al., 2018). The nervous system is composed of the central nervous system (CNS) and the peripheral nervous system (PNS). The CNS is the main part of the nervous system and is composed of the spinal cord and brain, which play a part in tumor development. Studies have reported that chronic stress causes disorders in the CNS and promotes tumor initiation and progression (Saul et al., 2005; Goldfarb et al., 2011; Levi et al., 2011; Partecke et al., 2016). *β*-adrenergic activation by the cAMP–PKA signaling pathway, e.g., *β*-adrenergic receptors (ARs) stimulated by norepinephrine (NE), is a major mechanism by which stress can enhance tumor development and increase vascularization (Thaker et al., 2006). Furthermore, immune regulation also plays a vital role in stress-induced tumor development (Dhabhar, 2009). Studies have shown that chronic stress causes a large-scale alteration of immune cells in tissues and induces suppression or dysregulation of immune function (Dhabhar et al., 2012; Dhabhar, 2014). The PNS emanates from the CNS and is divided into the autonomic systems and somatosensory, which are responsible for communicating with all parts of the body. Autonomic nerve density was reported to be associated with tumor prognosis and progression (Silverman et al., 2021). In terms of mechanism, infiltrating sympathetic nerves facilitated tumor progression and invasion through *β*-adrenergic activation (Pon et al., 2016). Adrenergic nerves also facilitate tumor growth by releasing NE to stimulate angiogenesis via VEGF signaling (Zahalka et al., 2017). In light of neural receptors are expressed by many immune cell types, it follows that immune cells may also participate in oncogenesis through adrenergic signaling (Silverman et al., 2021). Neuroendocrine factors contain neurotransmitters, neuropeptides and many other factors secreted by the nervous system mediate stimulatory or inhibitory functions by binding to their respective receptors. In recent decades, many discoveries have elucidated their regulatory roles in tissues and organs (Jiang et al., 2020a). For instance, studies in nerve growth factor (NGF) has reported that this factor acts not only on the PNS and CNS but also on nonneuronal and cancer cells (Aloe et al., 2016). NGF and its receptor TrkA have been implicated in the development of many aggressive cancers. However, NGF pathway has been proved to be critical to inflammation control and the immune response in tumor (Campos et al., 2007; Retamales-Ortega et al., 2017; Wehkamp et al., 2018; Triaca et al., 2019; Jiang et al., 2020b). Nerves regulate multiple components of the TME. Importantly, with the development of high-throughput sequencing techniques including single-cell RNA sequencing and spatial transcriptomics (Liu et al., 2022b), researchers found that immune and neuronal cells are often colocalized to form neuroimmune cell units (Huh and Veiga-Fernandes, 2020). And, both immune cells and neurons express receptors to sense neurotransmitters and cytokines, allowing direct interactions between the two systems (Seillet and Jacquelot, 2019).

Relatively few studies have investigated the functional role of neuroendocrine in carcinogenesis and regulation of the TME. Here, we summarize the mechanisms of neuroendocrine regulation of tissue-specific immunity based on the neuroendocrine signal transmission medium and how tissue-specific immune cells communicate with nerves by releasing different factors. We then focus on the role of neuroendocrine regulation in tumor immunity and how it informs prognosis and treatment.

## 2 Neuroendocrine regulation in tissue-specific immunity

The nervous system is composed of various components such as the CNS, sympathetic nerves, parasympathetic nerves, and sensory nerve nociceptors, and it mediates immune cells primarily by secreting neuroendocrine factors such as neurotransmitters and neuropeptides. These molecules can regulate tissue-specific immunity by binding to their corresponding receptors expressed on immune cells in various tissues. Here, we summarize the neuroendocrine regulation of tissue-specific immunity, categorized by signaling mediator.

### 2.1 Adrenergic regulation

The adrenergic system is composed of NE and epinephrine, which are the primary neurotransmitters secreted by postganglionic sympathetic neurons. They regulate cellular function through ARs, including the α1-, α2-, β1-, β2-, and β3-ARs (Tanner et al., 2021). The immune effects of adrenergic signaling are primarily transmitted by β2-ARs, which are expressed on nearly all major immune cell types (Marino and Cosentino, 2013). NE has been shown to mediate the cell trafficking and effector activities of immune cells *via* β2-AR.

NE affects the function of the CNS. Microglia are the most common resident innate immune cells in the CNS and monitor brain homeostasis by producing ligands that support neuronal survival, pruning non-functional synapses and removing dying neurons (Nayak et al., 2014; Werneburg et al., 2017). Studies have shown that NE upregulates the expression of the amyloid beta peptide (Aβ) receptor mFPR2 through activation of β2-AR in microglia, which helps to maintain the adequate uptake and clearance of Aβ(42). Moreover, increased secretion of NE suppresses the microglial response and reduces the upregulation of both anti- and pro-inflammatory cytokines through β2-adrenergic signaling (Lechtenberg et al., 2019). In general, NE can modulate microglial motility, which can affect the function of microglia in some pathogenic situations (Gyoneva and Traynelis, 2013; Umpierre and Wu, 2020).

Tissue-resident macrophages, ILCs, tissue-resident T cells and NK cells are common tissue-resident immune cells in the periphery. They act in a tissue-specific manner in adipose tissue, liver, lung and gut, where NE has been shown to modulate these cells via β2-adrenergic signaling. In adipose tissue, adipose tissue macrophages (ATMs) and ILC2s are common resident immune cells that act as dominant initiators of type 2 inflammation and tissue repair (Cording et al., 2016). Some studies have shown that sympathetic nerves maintain an anti-inflammatory state in mice by inhibiting TNF-α level in macrophages (Tang et al., 2015). Additionally, NE can promote extracellular fatty acid uptake and storage as triglycerides and reduce free fatty acid release from triglyceride-laden macrophages (Petkevicius et al., 2021a). However, another study found that the sympathetic nervous system exerts an indirect effect on ATMs through the modulation of adipocyte function instead of modulating the phenotype of ATMs directly (Petkevicius et al., 2021b). Similar findings were also found for ILC2s, in which PDGFRA + adipose stem cell (ASC) could serve as a messenger between the immune cells and nervous system. The sympathetic nerve can act on ASCs *via* β2-AR to control the level of glial-derived neurotrophic factor and indirectly regulate the activity of adipose tissue ILC2s via the neurotrophic factor receptor RET (50). Likewise, *β*-adrenergic signaling was also reported to regulate the production of IL-33 by a DPP4+PDGFRB + ASC subpopulation and enhance ILC2 accumulation indirectly (Shan et al., 2021).

In the intestine, tissue-specific immune cells include muscularis macrophages (MMs) and ILC2s. By studying the transcriptional profile, gut-innervating sympathetic neurons have been found to polarize MMs towards a protectively M2 phenotype through β2-AR (52). In murine models of enteric infections, MMs upregulate a neuroprotective program via β2-AR and constrain neuronal death through an arginase 1-polyamine axis (Matheis et al., 2020). ILC2s participate in multiple intestinal physiological processes, including tissue repair, metabolic homeostasis, allergic inflammation and host defense against infections (Cardoso et al., 2017). Research has shown that β2-AR deficiency results in exaggerated ILC2 responses and type 2 inflammation in the intestine (Moriyama et al., 2018). Another study also demonstrated that sympathetic innervation constrains the effects of innate immune responses on microbes in the gut through the β2-AR pathway (Willemze et al., 2019). β2-AR has been proven to mediate negative regulation of ILC2s through the inhibition of cell proliferation and effector function (Moriyama et al., 2018). A recent study reported that colonic sympathetic nerves can also exert an indirect effect on immune cells through endothelial MAdCAM-1. In murine models, activation of local sympathetic nerves decelerated colitis and reduced the abundance of immune cell (Schiller et al., 2021). Hepatic invariant NKT (iNKT) cells are tissue-specific immune cells that primarily reside in the liver (Bendelac et al., 2007). One study showed that the immunosuppressive function of iNKT cells was mediated through NE (59).

**FIGURE 1:**
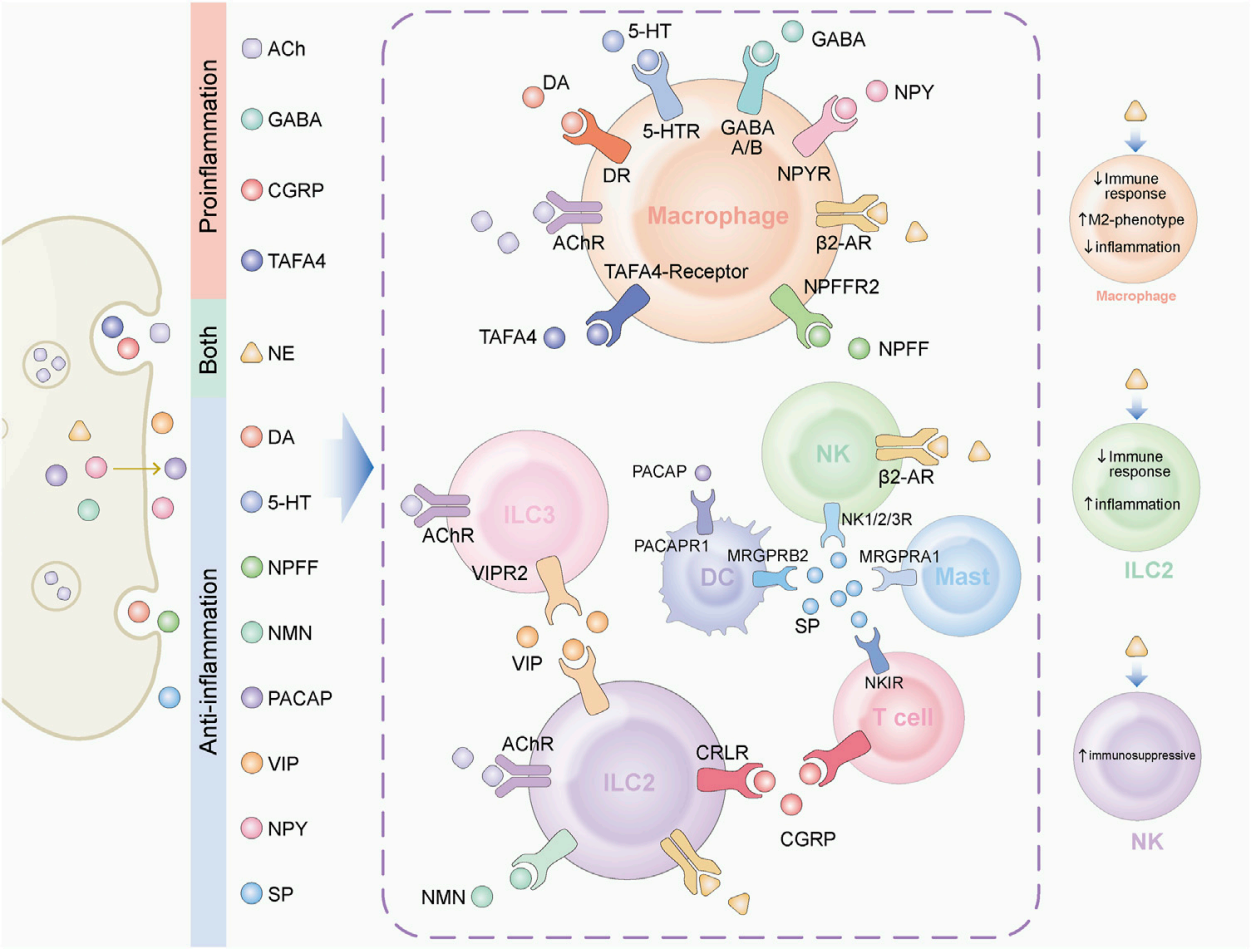
Neuroendocrine factors play various role in tissue immune regulation. A variety of neuroendocrine factors play regulatory roles in tissue-resident immune cells. The target cells and their receptors for different factors are displayed in the figure. On the left, neuroendocrine factors are classified according to their function. ACh, GABA, CGRP and TAFA4 mainly play a pro-inflammatory function. DA, 5-HT, NPFF, NMU, PACAP, VIP, NPY, and SP mainly play an anti-inflammatory function. NE plays different roles in different target cells. On the right side of the figure, we marked the functions of NE.

In summary, adrenergic signaling regulates a variety of tissue-resident immune cells in the CNS and PNS in which plays an anti-inflammatory and immunosuppressive role (Figure 1, Table 1). Thus, adrenergic regulation is an important neuroendocrine modulating method for tissue-specific immunity.

**TABLE 1:** Summary of central and peripheral neuroendocrine factors regulating tissue specific immunity.

Ligand	Target tissue	Target cell	Receptor	Effect
NE	CNS	Microglia	β2-AR	↑ Aβ clearance (Kong et al. (2010)
				↓ Microglia response (Lechtenberg et al. (2019)
	Adipose tissue	ATM	β2-AR	↓ Inflammation (Tang et al. (2015)
				↓ Extracellular fatty acid (Petkevicius et al. (2021a)
				indirect effect through adipocyte (Petkevicius et al. (2021b)
		ILC2	β2-AR	Indirect effect through ASC (Cardoso et al. (2021); Shan et al. (2021)
	Intestine	MM	β2-AR	↑ M2 phenotype (Gabanyi et al. (2016)
				↑ Neuroprotection (Matheis et al. (2020)
		ILC2	β2-AR	↑ Type 2 inflammation (Moriyama et al. (2018)
				↓ Innate immune response (Willemze et al. (2019)
		Immune	β2-AR	Indirect effect through endothelium (Schiller et al. (2021)
	liver	iNKT	β2-AR	↑ Immunosuppressive function (Bendelac et al. (2007); Wong et al. (2011)
ACh	CNS	Microglia	mAChR	↑Chemotaxis and phagocytosis (Pannell et al. (2016)
				↓ Inflammation (Wang et al. (2003); Li et al. (2019)
			α7nAChR	↑ M2 phenotype (Zhang et al. (2017)
				↑ Aβ clearance (Hoskin et al. (2019)
	Llung	ilc2	α7nAChR	↓Type 2 inflammation (Galle-Treger et al. (2016); Pavón-Romero et al. (2021)
	Liver	Macrophage	AChR	↑Phagocytosis and secretion (Fonseca et al. (2019)
				↑ FoxM1, ↑ liver regeneration (Izumi et al. (2018)
	Intestine	MM	α7nAChR	↓ Inflammation (Matteoli et al. (2014); Yang et al. (2021)
		ILC2	AChR	↓Type 2 inflammation (Chu et al. (2021)
		ILC3	AChR	↑ PCTR biosynthetic pathway (Dalli et al. (2017)
	Spleen, pancreas	Macrophage	α7nAChR	↓ Inflammation (Zhang et al. (2020)
DA	CNS	Microglia	D1R	↑ Microglial migration (Stolero and Frenkel (2021)
			D2R	↑ ROS and NO (Stolero and Frenkel (2021)
				↑Inflammation (Stolero and Frenkel (2021)
			DR	↑ Extracellular trap in microglia (Agrawal et al. (2021)
5-HT	CNS	Microglia	5-HTR	↑Inflammation ↑IL-6 (Quintero-Villegas and Valdes-Ferrer, 2019)
GABA	CNS	Microglia	GABA_A_/GABA_B_	↓ Microglial neurotoxicity (Lee (2013); Crowley et al. (2016)
				↓ Inflammation (Lee (2013); Crowley et al. (2016)
NPFF	Adipose tissue	ATM	NPFFR2	↑ M2 phenotype (Waqas et al. (2017)
				↑ATM proliferation (Waqas et al. (2017)
NMU	Lung	ILC2	NMUR1	↑Type 2 inflammation (Wallrapp et al. (2017); Chen et al. (2021)
				↑γδ T cell, ↑IL-17A (Wallrapp et al. (2017); Chen et al. (2021)
	Intestine	ILC2	NMUR1	↑Type 2 inflammation (Klose et al. (2017)
				↑ IL-10 (Bando et al., 2020)
PACAP	Skin	DC	PACAPR1	↑ Contact hypersensitivity (Yamamoto et al., 2021)
				↑ CCR7, ↑ CXCR4 (Yamamoto et al., 2021)
VIP	Lung	ILC2	VIPR2	↑Type 2 inflammation (Talbot et al. (2015); Seillet and Jacquelot (2019))
	Intestine	ILC3	VIPR2	↑ IL-22, ↑ barrier function of the epithelium (Seillet et al. (2020); Talbot et al. (2020))
NPY	CNS	Microglia	NPYR	↑ Inflammation, ↓ microglial activation and phagocytosis (Gonçalves et al. (2012); Pain et al. (2019); Chen et al. (2020)
	Spleen	Immune	NPYR	↓ Immune responses (Yu et al. (2022)
CGRP	Skin, lung and intestine	ILC2	CRLR	↓ Type 2 inflammation (Wallrapp et al. (2019); Xu et al. (2019)
				↑ IL-5, ↓IL-13 (Nagashima et al. (2019)
	Lung	T cell	CRLR	↓ protective immunity (Baral et al. (2018); Pinho-Ribeiro et al. (2018)
				↓ γδ T cell number (Baral et al. (2018); Pinho-Ribeiro et al. (2018)
				↓ Neutrophil (Baral et al. (2018); Pinho-Ribeiro et al. (2018)
CGRP (PNEC)	Lung	ILC2	CRLR	↑Type 2 inflammation (Sui et al. (2018)
				↑ ILC2 activity (Sui et al. (2018)
SP	Intestine	NK cell	NK1R/NK2R/NK3R	↑ Inflammation (Talbot et al. (2015); Seillet and Jacquelot (2019)
	Lung	T cell	NK1R	↑ T helper 2 cell influx and polarization (Crosson et al. (2021)
	Skin	DC	MRGPRA1	↑ DC migration, ↑ T helper 2 cell differentiation (Perner et al. (2020)
				↑ IL-23, ↑neutrophil, ↑monocytes (Riol-Blanco et al. (2014)
				↑ Inflammation (Riol-Blanco et al. (2014)
		Mast cell	MRGPRB2	↑ Mast cells degranulation (Serhan et al. (2019)
				↑ Inflammation (Serhan et al. (2019)
TAFA4	Skin	Macrophage	unknown	↑ IL-10, ↓ inflammation (Hoeffel et al. (2021)

DC, dendritic cell; ILC, innate lymphoid cell; CNS, central nervous system; NE, norepinephrine; AR, adrenergic receptor; ATM, adipose tissue macrophage; ASC, adipose stem cell; MM, muscularis macrophage; iNKT, invariant natural killer T; ACh, acetylcholine; mAChR: metabotropic muscarinic ACh, receptor; nAChR: ionotropic nicotinic ACh, receptor; 5-HT: serotonin; GABA, gamma-aminobutyric acid; DA, dopamine; DR, dopamine receptor; 5-HTR: 5-HT, receptor; NMU, neuromedin U; SP, substance P; CGRP, calcitonin gene-related peptide; PNEC, pulmonary neuroendocrine cell; PACAP, pituitary adenylate cyclase-activating polypeptide; VIP, vasoactive intestinal peptide; NPFF, neuropeptide FF; NPY, neuropeptide Y; CRLR, receptor calcitonin receptor like receptor; MRGPR, Mas-related G-protein coupled receptor member.

### 2.2 Cholinergic regulation

The cholinergic system, which is found in both neuronal and nonneuronal cells, is a network that performs various complex functions in the body. It is composed of acetylcholine (ACh), cholinergic receptors (AChRs), acetylcholinesterase enzyme and choline acetyltransferase enzyme (Halder and Lal, 2021). ACh is the classical neurotransmitter in the cholinergic system. The receptor of ACh is divided into ionotropic nicotinic ACh receptors (nAChRs) and metabotropic muscarinic ACh receptors (mAChRs).

ACh affects the function of the CNS. AChRs are expressed on microglia in the CNS. mAChR stimulation modulates microglial chemotaxis and phagocytic activity via IFN-γ activation (Pannell et al., 2016). Regarding nAChRs, α7nAChR is an essential regulator of inflammation (Wang et al., 2003). The anti-inflammatory ACh response in microglia is mediated through α7nAChR. This response helps to lower inflammatory cytokine levels and microglial activation (Li et al., 2019). Similarly, ACh inhibits LPS-induced IL-1β and IL-6 elevation and promote IL-4 and IL-10 production through α7nAChRs to promote the M2 phenotype (Zhang et al., 2017). In addition, ACh stimulation of α7nAChR in microglia also enhances Aβ clearance (Hoskin et al., 2019).

Tissue-resident macrophages and ILCs are common tissue-resident immune cells in the periphery. They act in a tissue-specific manner in the liver, lung, gut, where ACh has been shown to modulate these cells *via* ACh-AChR signaling. In the lung, ILC2s are common resident immune cells. The ACh-α7nAChR axis in ILC2s decreases the synthesis of TNF-α, IL-1, and IL-6 (66). However, ILC2 transcription factor GATA-3 and the inflammatory modulator NF-κB is diminished. In general, ACh-α7nAChR signaling in ILC2s helps to promote anti-inflammatory function (Galle-Treger et al., 2016). In the liver, hepatic macrophages are the common resident immune cells. The vagus nerve regulates the secretory and phagocytic activity of resident macrophages *via* cholinergic signaling (Fonseca et al., 2019). ACh-AChR signal-mediated IL-6 production in hepatic macrophages upregulates the expression of FoxM1 in hepatocytes, leading to liver regeneration (Izumi et al., 2018). In the intestine, tissue-specific immune cells include MMs, ILC2s and ILC3s. The vagus nerve regulates the anti-inflammatory effect of intestinal MMs via α7nAChR-mediated JAK2/STAT3 signaling pathway (Matteoli et al., 2014; Yang et al., 2021). The ACh-AChR axis in ILC2s also causes anti-inflammatory effects by promoting ILC2 cytokine production (Chu et al., 2021). Moreover, AChs upregulate the PCTR biosynthetic pathway in ILC3s(Dalli et al., 2017). Besides, the vague nerve is also reported to modulate anti-inflammatory effect through the α7nAChR on macrophages in spleen and pancreas (Zhang et al., 2020).

In summary, cholinergic signaling facilitates the behavior of tissue-resident macrophages and promotes the anti-inflammatory function of ILC2s in the nervous system (Figure 1, Table 1). Thus, cholinergic regulation is another important neuroendocrine modulating method for tissue-specific immunity.

### 2.3 Regulation *via* other neurotransmitters

In addition to NE, epinephrine and ACh, neurotransmitters also include dopamine (DA), serotonin (5-HT) and gamma-aminobutyric acid (GABA). Research on the neuroendocrine regulation of immune cells by these other neurotransmitters has mainly focused on microglia. Microglia express several receptors for DA, 5-HT and GABA (Younger et al., 2019; Stolero and Frenkel, 2021).

DA is a neurotransmitter synthesized in both the CNS and PNS. It is mainly distributed in the CNS and has been detected in the liver, spleen, pancreas and gut (Peters et al., 2014; Klein et al., 2019). Dopamine receptors (DRs), including D1R, D2R, D3R, D4R, and D5R, are present on microglia and some other macrophages (Thomas Broome et al., 2020). DA promotes microglial migration, increases NO and ROS production through D1R, and the secretion of proinflammatory cytokines, such as IL-1β and IL-6 through D2R (75). Also, DA can induce the formation of DNA-based extracellular traps in microglia which participate in immunosurveillance and pathogen clearance (Agrawal et al., 2021) (Figure 1, Table 1).

5-HT, a biogenic amine synthesized from tryptophan, is a well-known neurotransmitter in the CNS which plays a critical roles in mood stability, sleep patterns and pain tolerance. The effects of 5-HT are regulated through 5-HT receptors. It also exist as an important signaling molecule in the periphery, especially in the gastrointestinal tract (Ye et al., 2021). 5-HT plays a role in CNS inflammation and repair. Administering 5-HT to microglia induces inflammatory initiation and IL-6 production (Quintero-Villegas and Valdés-Ferrer, 2019) (Figure 1, Table 1).

GABA is known as a main inhibitory amino acid neurotransmitter in the CNS. The physiological roles of GABA are related to the modulation of synaptic transmission. Two distinct classes of GABA receptors have been identified, including ionotropic receptor GABA_A_ and metabotropic receptor GABA_B_ (Ngo and Vo, 2019). Microglia are known to express both GABA_A_ and GABA_B_. GABA has been shown to decrease microglial neurotoxicity and exert an anti-inflammatory effect (Lee, 2013; Crowley et al., 2016) (Figure 1, Table 1).

There are few studies on the role of these neurotransmitters in peripheral tissue-specific immunity. However, there is evidence for their presence in the periphery; for instance, the airway epithelium contains pulmonary neuroendocrine cells filled with 5-HT and GABA neuropeptides (Magnon, 2021). Some peripheral tissue-specific immune cells have been reported to express corresponding neurotransmitter receptors, providing the possibility for neuroendocrine interactions between them.

### 2.4 Regulation *via* neuropeptides

Neuropeptides are peptide substances produced by neurons that play key roles in modulating cell function by binding to specific receptors. Examples of neuropeptides include neuromedin U (NMU), neuropeptide calcitonin gene-related peptide (CGRP), substance P (SP), neuropeptide TAFA4, pituitary adenylate cyclase-activating polypeptide (PACAP), vasoactive intestinal peptide (VIP), neuropeptide FF (NPFF), neuropeptide Y (NPY) and somatostatin (SST). Neuropeptides and their receptors have regionally restricted distributions in the nervous system (Hoyer and Bartfai, 2012). Transcriptomic analyses have indicated that immune cells express numerous neuropeptide receptors (Seillet and Jacquelot, 2019). Thus, neuropeptides may play a vital role in mediating tissue-specific immunity.

#### 2.4.1 Neuropeptides secreted by the central nervous system and autonomic nerves

NPFF is a CNS octapeptide that plays a part in pain modulation and opiate tolerance. NPFF has two receptors, NPFF receptor 1 and 2 (NPFFR1, NPFFR2) (Bonini et al., 2000). In peripheral tissues, only NPFFR2 is expressed in ATMs (Waqas et al., 2017). NPFF promotes M2 phenotype and increase the proliferation of ATMs. Specifically, NPFF suppressed the expression of the E3 ubiquitin ligase RNF128, which promote the stability of phosphorylated STAT6 and increased expression of gene related to M2 macrophage. NPFF induced ATM proliferation accompanied with the increase of NDRG2 expression and suppression of MAFB expression (Waqas et al., 2017) (Figure 1, Table 1).

NMU is a neuropeptide widely distributed in the human body and has two receptors, NMU receptor 1 and 2 (NMUR1, NMUR2). NMU mainly functions on ILC2s through NMUR1 (Martinez and O'Driscoll, 2015). NMU stimulates ILC2 activation and promotes type 2 cytokine responses that can induce antimicrobial and inflammatory responses (Klose et al., 2017). In the intestine, NMU increases IL-10 production in intestinal ILC2s. However, the function of ILC2-derived IL-10 in the intestine remains unknown (Bando et al., 2020). NMU can also amplify lung inflammation driven by ILC2s. IL-9 derived from ILC2 plays an important role in increasing the abundance of γδ T cell and IL-17A production (Wallrapp et al., 2017; Chen et al., 2021).

PACAP and VIP are two highly related neuropeptides widely distributed in organisms and have immunomodulatory actions. The receptors of PACAP and VIP are expressed by numerous immune cell types (Abad and Tan, 2018). The neuropeptide PACAP is an antimicrobial peptide induced in the brain in response to bacterial and fungal infection (Lee et al., 2021). PACAPR1 is a main receptor of PACAP expressed on DC. PACAP induces cutaneous DC functions and promotes the development of contact hypersensitivity through PACAPR1. The expression of CCR7 and CXCR4 of DCs was enhanced by PACAP *in vitro* (Yamamoto et al., 2021).

NPY is a neuropeptide widely distributed in the human body and is secreted mainly by the CNS and sympathetic nerves, where it is co-released with NE. NPY exerts its effects through interacting with NPY receptors (NPYRs), among which NPY1R is the most abundantly expressed NPYR receptor in immune cells (Dimitrijevic and Stanojevic, 2013). NPY inhibits microglial activation and phagocytosis and affects the direction of migration and phagocytosis. NPY also inhibits cytokine secretion of microglia, especially the secretion of proinflammatory factors (Gonçalves et al., 2012; Pain et al., 2019; Chen et al., 2020). Besides, NPY was reported to attenuate the splenic immune response as well (Yu et al., 2022) (Figure 1, Table 1).

Regarding VIP, intestinal ILC3s and pulmonary ILC2s highly express VIP receptor 2 (VIPR2). In the intestine, VIP activates ILC3s, enhancing the production of IL-22 and the barrier function of the epithelium (Seillet et al., 2020). This dynamic neuroimmune circuit in the intestine is regulated by feeding and circadian mechanisms (Talbot et al., 2020) (Figure 1, Table 1).

SST is a neuropeptide with generally inhibitory function which commonly produced by endocrine cells and the CNS(Weckbecker et al., 2003). SST mediates its biological functions via SST receptors (subtype 1–5), which was found expressing in multiple immune cells (Møller et al., 2003). However, existing studies have shown that SST only affects immune cells in peripheral blood of normal tissues. For instance, SST reduced the secretion of INF-γ from peripheral blood mononuclear cell and inhibit the production of immunoglobulin by B lymphocytes. Also, the chemotaxis of peripheral blood monocytes is inhibited by this peptide (Pintér et al., 2006).

#### 2.4.2 Neuropeptides secreted by sensory nerves

Nociceptor sensory neurons are responsible for the detection of potentially damaging stimuli and elicit defensive behaviors (Chiu et al., 2013). The neuropeptides CGRP and SP are nociceptor mediators that act in the skin, lung and intestine. TAFA4 is a neuropeptide secreted by sensory neurons in skin. VIP mentioned above can also be secreted by nociceptors. Tissue-resident macrophages, ILC2s, NK cells, DCs and γδ T cells are common tissue-resident immune cells reside within epithelial layers of the lung, skin, and gut (Zheng et al., 2013).

CGRP regulates a variety of immune cells which express the receptor, calcitonin receptor-like receptor (CRLR). Broadly speaking, CGRP is a negative regulator of ILC2 responses, which is necessary for suppressing ILC2 expansion and maintaining homeostasis of the type 2 immune machinery (Wallrapp et al., 2019; Xu et al., 2019). Additionally, CGRP in concert with NMU is reported to promote IL-5 but constrain IL-13 expression (Nagashima et al., 2019). In the lung, nociceptors suppress protective immunity through the release of CGRP. Specifically, CGRP suppressed the recruitment and surveillance of neutrophils (Pinho-Ribeiro et al., 2018), and reduced lung γδ T cell numbers, which act as first responders to infection (Baral et al., 2018). On the contrary, pulmonary neuroendocrine cells (PNECs) are a kind of rare airway epithelial cells that reside near airway branches. CGRP secreted by PNECs enhances ILC2 activity and the production of cytokines (Sui et al., 2018).

In the intestine, precursor and immature NK cells exhibit tissue-resident signatures (Dogra et al., 2020). SP is released from nociceptors after stimulation through the neurokinin receptors NK1R, NK2R and NK3R and has been shown to regulate cell migration and proliferation to generate neurogenic inflammation (Seillet and Jacquelot, 2019). In the airways, lung nociceptor neurons release SP, and amplify T helper 2 cell influx and polarization via NK1R (Crosson et al., 2021). In skin, SP interacts with DCs and mast cells through the Mas-related G-protein coupled receptor member (MRGPR). Sensory neurons release SP, which acts through MRGPRA1 to induce CD301b+ DC migration to the draining lymph node (dLN). Migrated DCs initiate T helper-2 cell differentiation in the dLNs(Perner et al., 2020). One study reported that nociceptors induce the recruitment of neutrophils and monocytes, driving skin inflammation. Nociceptors promote the production of IL-23 by dermal DCs. IL-23 then acts on IL-23R+ γδT17 cells to promote the secretion of IL-17F and IL-22 (119). Nociceptors can also amplify skin inflammation by inducing the degranulation of mast cells that are contiguous to the nociceptors and activating MRGPRB2 on the mast cells (Serhan et al., 2019).

TAFA4 is a neuropeptide secreted by sensory neurons in skin. TAFA4 reduce inflammation and cell infiltration by inducing IL-10 production from Tim4+ dermal macrophages (Flayer and Sokol, 2021; Soler Palacios and Gutiérrez‐González, 2022). TAFA4 can also affect the inflammatory mechanisms of other macrophage subsets, including CD206+ dermal macrophages and peritoneal macrophages (Hoeffel et al., 2021). In the lung, nociceptors can induce the production of VIP which in turn stimulates ILC2s to promote the occurrence of inflammation (Talbot et al., 2015; Seillet and Jacquelot, 2019).

In a word, sensory nerves function primarily in their widely distributed areas including skin, lung and gut. Neuropeptides secreted by sensory neurons play diverse roles in immune responses against various pathogens (Figure 1, Table 1).

## 3 Tissue-specific immune effects on nerves

Tissue-specific immune cells regulate nerves by releasing various factors such as cytokines, chemokines, small-molecule peptides and neurotrophic factors.

Among the tissue-specific immune cells, tissue-resident macrophages exhibit a rich regulatory role in different tissues. In adipose tissue, ATM expresses neurotrophic factors to promote white adipose tissue innervation (Xie et al., 2022). Also, ATM reportedly controls brown adipose tissue innervation (Wolf et al., 2017). In subcutaneous fat, a new subset of immune cells called cholinergic adipose macrophages (ChAMs) has been identified. ChAMs secrete ACh to regulate thermogenic activation via β2-AR (Knights et al., 2021). ATM can also mediate sympathetic neurons through ROBO1 receptor. Slit3 is a macrophage cytokine secreted by ATM. It binds to the ROBO1 receptor to stimulate Ca2+/calmodulin-dependent protein kinase II signaling and NE release, which enhances adipocyte thermogenesis (Wang et al., 2021). Additionally, MMs regulate peristaltic activity of the colon by secreting bone morphogenetic protein 2 (BMP2), which activates the expression of BMP receptor on enteric neurons. Enteric neurons, in turn, secrete a growth factor required for macrophage development called colony stimulatory factor 1 (CSF1), (Muller et al., 2014). Sympathetic neuron-associated macrophages are another previously undescribed population of resident macrophages that mediate NE clearance via SLC6A2 and MAOA expression (Pirzgalska et al., 2017). A nerve- and airway-associated macrophage (NAM) subset has been identified in the lung. Their closely associated nerves have sympathetic fibers. However, the relationship between NAMs and their associated nerves is unclear (Ural et al., 2020).

In the CNS, microglia perform negative feedback control of neuronal activity. The suppression of neuronal activation depends on the ability to sense and catabolize extracellular ATP of microglia (Badimon et al., 2020). ILC2s also play a part. In the lung, nociceptors can sense IL-5 released by activated immune cells, and IL-5 further induces VIP production. VIP then stimulates ILC2s, creating an inflammatory signaling loop that promotes allergic inflammation (Talbot et al., 2015; Seillet and Jacquelot, 2019). ACh is a broadly distributed signaling molecule that is not only produced by neurons, but also by numerous immune cells. Several immune cell types respond to ACh signaling and can also produce ACh directly (Cox et al., 2020).

## 4 Neuroendocrine tissue-specific immunity axis in cancer

It is generally accepted that tumor innervation correlates with tumor progression across multiple solid tumor types (Albo et al., 2011; Huang et al., 2014; Kappos et al., 2018; Renz et al., 2018). Specifically, autonomic nervous infiltration was discovered to affect the development and dissemination of breast cancer, head and neck carcinoma, prostate cancer, liver cancer, lung cancer, pancreatic cancer, cholangiocarcinoma, colorectal cancer, ovarian cancer, glioma and gastric cancer (Magnon et al., 2013; Zhao et al., 2014; Partecke et al., 2016; Pon et al., 2016; Zahalka et al., 2017; Faulkner et al., 2019; Sha et al., 2019). While sensory neurons were found to play a part in tumor development of melanoma and pancreatic cancer (Saloman et al., 2016; Prazeres et al., 2020). Meanwhile, high intra-tumoral nerve density is associated with poor prognosis and high recurrence in multiple cancers (Silverman et al., 2021). Studies have shown that the mechanism by which tumor innervation affects cancer can be divided into three parts: direct stimulation of tumor proliferation, promotion of vascularization, and indirect modulation of tumors through immune changes. In this context, we discuss the effect of neuroendocrine regulation on tissue-specific immunity in the TME.

In breast cancer, sympathetic innervation accelerates tumor progression, while parasympathetic innervation decelerates tumor progression in the TME (26). The sympathetic nervous system affects stress-induced cancer behaviors, including initiation, progression and metastasis, by modulating tumor-associated immune cells via the NE-βAR signaling pathway in four ways. First, *β*-adrenergic signaling represses an effector phenotype of CD8^+^ T cells in the TME. Reducing *β*-adrenergic signaling induced an immunologically active TME in tumor-bearing animal models (Bucsek et al., 2017). Second, parasympathetic innervation constrains the function of immune checkpoints. Sympathetic nerve denervation and parasympathetic neurostimulation reduced the level of PD-1, PD-L1, and FOXP3 on CD4^+^ or CD8^+^ T cells in murine models of breast cancer. This effect was also proved in human breast cancer samples (Kamiya et al., 2019). Next, the NE-βAR signaling pathway increased the infiltration of macrophages in the TME and induced the differentiation to a prometastatic M2 macrophage phenotype. Stress-induced NE-βAR signaling activation induced a metastasis to distant tissues in mouse breast cancer models (Sloan et al., 2010). Finally, NE-βAR signaling pathway promotes breast cancer metastasis by recruiting myeloid-derived suppressor cells (MDSCs). MDSCs are a population of cells with immunosuppressive phenotype. In murine models of breast cancer, chronic stress leaded to the elevation of MDSCs, accelerated breast cancer metastasis, and upregulated IL-6 expression and JAK/STAT3 signaling pathways (an et al., 2021). Another study reveals that the expression level of β2-AR on MDSCs increases with tumor growth. β2-adrenergic signaling increases oxidative phosphorylation, increase fatty acid oxidation, decreases glycolysis, and also increases autophagy and activates the arachidonic acid cycle (Mohammadpour et al., 2021). A recent study revealed that in brain metastases from breast cancer, neuronal exposure induces synaptic mediators and neurotransmitter signaling in tumors (Deshpande et al., 2022). This research suggests that neuroendocrine regulation in brain metastases seems to be more direct, and whether immune cells in brain metastases TME are regulated by neuroendocrine factors might be the direction of future research.

Likewise, in hepatocellular carcinoma, *β*-adrenergic signaling promotes tumor growth by mobilizing MDSCs to tumor tissues. In the hepatocellular carcinoma mouse models, stress enhanced tumor progression. Specifically, *β*-adrenergic signaling changed the spleen structure, and caused a redistribution of MDSCs to tumors. Also, the author found that splenectomy could constrain tumor growth and prevent increasement of macrophages in tumor tissues in stressed mice (Jiang et al., 2019). Another study pointed out the possible mechanism of this mobilization. The recruitment of MDSCs was modulated through *β*-adrenergic-activated CXCL5-CXCR2-Erk signaling cascades. Chronic stress upregulated the expression of CXCR2 and pErk1/2 in MDSCs, and the expression of CXCL5. *In vitro*, T-cell proliferation was obviously constrained in NE treated medium. *In vivo*, *β*-adrenergic blockade reversed the acceleration of tumor growth induced by chronic stress and CXCL5-CXCR2-Erk signaling pathway was suppressed (Cao et al., 2021).

Moreover, in prostate cancer, the density of sympathetic fibers in tumors is associated with poor clinical outcomes (Magnon et al., 2013). It was observed that prostate cancer patients with depression showed higher tumor-associated macrophage infiltration. NPY is co-released with NE in sympathetic nerves. Animal experiments revealed that NPY released from NE-treated prostate cancer cells promotes macrophage trafficking and IL-6 release, which subsequently activates the STAT3 signaling pathway (Cheng et al., 2019). Analyses of clinical prostate cancer samples have also suggested that elevated NPY promotes prostate cancer development and is associated with poor prognosis and therapy resistance (Rasiah et al., 2006; Ding et al., 2021). Additionally, NK cells are mobilized by epinephrine through the *β*-adrenergic signaling pathway, which can reduce the tumor initiation and recurrence. In mouse tumor models, epinephrine induced selective mobilization of IL-6-sensitive NK cells while IL-6 inhibited the infiltration and activation of NK cells in TME (Pedersen et al., 2016).

Yet, different perspectives have been raised in pancreatic cancer. Chronic stress is observed to increase pancreatic cancer progression and can be antagonized by *β*-AR blockade in some studies (Partecke et al., 2016; Renz et al., 2018). However, a recently published study demonstrated that sympathetic nerves ablation increases tumor growth and spread by increasing intra-tumoral CD163+ macrophage numbers. In the mouse model of pancreatic cancer, the sympathetic nerves exert a protective function during the early stage of tumor. The author mentioned that the converse response to sympathectomy in pancreatic cancer may be shaped by the particular TME (Guillot et al., 2022). Otherwise, ablation of sensory neurons of pancreatic cancer reportedly slows initiation and progression of cancer. However, the study did not mention its impact on tumor immunity (Saloman et al., 2016).

Sensory innervation was reported to constrain melanoma progression. Ablation of sensory nerves lead to worse outcomes in melanoma-bearing mice (Prazeres et al., 2020). In a recent study, Costa et al. (2021) informs that sensory neuron activity may slow the melanoma progression by inducing tumor immunosurveillance. Specifically, in sensory neuron-overactivated melanoma mice, the number of MDSCs and neutrophils significantly decreased, while tumor-infiltrating DCs, CD8 + T cells, CD4 + T cells, γδ T cells and NK cell was detected to increase. The expression of immune checkpoint molecules such as PD-1 and CTLA-4 decreased. And it induces a Th17-immune response in the melanoma microenvironment. Furthermore, the author validated these results in a large human melanoma cohort. They found that SCN10A (encoding Nav1.8), a key gene of sensory neurons, has higher expression in patients with better prognosis. The enrichment of tumor-infiltrating immune cells showed an increase of DCs, CD8 + T cells, CD4 + T cells and NK cell in patients with better prognosis. Conversely, an earlier published study by Keskinov et al. (2016) reported that the dorsal root ganglia (DRG) of sensory nerves contribute to melanoma progression by recruiting MDSCs which help to create an oncogenic TME. Experimental results suggested that, *in vitro*, melanoma cells can activate DRG neurons and increase the expression of chemokines that attract MDSC. *In vivo*, in the presence of DRG cells, tumor growth was accelerated and the number of MDSC increased. To sum up, for sensory neuron modulation, Costa et al. (2021) used a designer drug to selectively activate or inhibit sensory neurons within the tumor *in vivo*, whereas Keskinov et al. (2016) used DRG cells injection in mice. Besides, Costa et al. (2021) focused on multiple kinds of cells involved in tumor immunity including T cells, DCs, and MDSCs, while Keskinov et al. (2016) focused only on the alteration of MDSCs. Furthermore, Costa et al. (2021) proved their findings through *in vivo* assays and bioinformatic validations, while Keskinov et al. (2016) validated through *in vitro* and *in vivo* assays. In our opinion, there are several possible reasons for the opposite results produced by the two studies. First, the nerve fibers at work are different. It speculates that it is inappropriate to choose DRG cells as a representation of sensory neurons. The DRG is an enlargement of the dorsal root that houses somata of sensory neurons. But DRGs can also receive signals from the autonomic nervous system by connecting to the sympathetic nerve via rami communicantes nerves (Esposito et al., 2019). Consequently, the tumor-promoting effects of DRGs mentioned in the study of Keskinov et al. (2016) is likely to be partially mediated by the autonomic nerves. The oncogenic role of the autonomic nervous system in multiple cancers has been mentioned above. Apart from that, the neuroendocrine factors at work are different. Signals from sensory neurons in normal tissues can be transmitted to immune cells through various factors such as CGRP, SP, and VIP. But neither study involved exploration on factors that signal between neurons and immune cells. Although Costa et al. (2021) observed an elevation of CGRP level after sensory nerve activation, this mechanism has not been confirmed. Whether sensory nerves also interact with immune cells through these substances in the melanoma microenvironment might be a direction of future research. We believe that further explorations of neuroendocrine factors involved in immune regulation in melanoma may answer the reasons for the opposite conclusions of the two studies above.

An analysis of clinical samples reveals that GABA is associated with poor prognosis in lung cancer and colon adenocarcinoma (Huang et al., 2022). GABA is reported to have an anti-tumor immunity function through two different pathways. One study informed GABA promotes the differentiation from monocyte to anti-inflammatory macrophages that secrete IL-10. GABA also inhibit CD8^+^ T cell killer function (Zhang et al., 2021). Another study suggested GABA activates the GABA_B_ receptor to stimulate tumor cell proliferation and suppress CD8^+^ T cell infiltration in TME. In mouse models, targeting GABA_B_ overcomed resistance to anti-PD-1 immune checkpoint blockade therapy (Huang et al., 2022).

Regarding neuropeptides, a systematic review found evidence that the elevation of SP and NK-1R are oncogenic events in head and neck carcinogenesis and probably act in the early stages of tumorigenesis (Gonzalez-Moles et al., 2021). Proline rich polypeptide 1, (PRP-1) is a neuropeptide secreted by the brain neurosecretory cells which causes inhibition of chondrosarcoma cell growth (Galoyan, 2000; Galoian et al., 2009). A study proves that PRP-1 functions via toll like receptor family TLR1/2, TLR6 and mucin MUC5B which are responsible for innate immunity pattern recognition. This result suggests that the anti-tumor effect of PRP-1 might be related to immune cells in TME. But the author also suggested that the receptors predominantly located in the tumor nucleus (Galoian et al., 2018). Another research demonstrated that PRP-1 can also inhibit cancer stem cell proliferation in chondrosarcoma (Granger et al., 2020). In addition, somatostatin analog (SSA) is the analog of SST which is now widely used in the treatment of neuroendocrine neoplasms. Long-acting repeatable of SSA decrease the level of total regulatory T cells and MDSCs, as well as the expression of PD1, CTLA4 and ENTPD1 in neuroendocrine neoplasms which exert an anti-tumor immunosurveillance function (von Arx et al., 2020). SSA was also reported to decrease cell proliferation and tumor growth in multiple cancers, such as lung cancer, breast cancer, colon carcinoma and endometrial carcinomas (Kahán et al., 1999; Engel et al., 2005; Treszl et al., 2009; Hohla et al., 2010). However, if these oncogenic effects are associated with tumor-associated immune cells has not yet been discussed. The function of neuropeptides in tumor-specific immunity requires further research.

In brief, under the regulation of neuroendocrine factors, some components of TME play the role of immunosurveillance, such as effective T cells, NK cells, and the immune checkpoint molecules produced by them. Yet some other components play the opposite pro-tumor effects including MDSCs, regulatory T cells and tumor-associated macrophages. Immunosurveillance effects are negatively regulated by *β*-adrenergic signaling in breast, prostate and liver cancers. In lung and colon cancer, immunosurveillance is negative regulated by sensory nerves. Pancreatic cancer immunosurveillance is positive regulated by *β*-adrenergic signaling, however negative regulated by sensory nerves. Melanoma is mainly mediated by sensory nerves, but the direction of regulation is controversial. Lastly, immunosurveillance in neuroendocrine neoplasm is positive regulated by SST.

From the perspective of therapy, there are also many researches emerged in recent years. Since tumor innervation plays an active role in cancer initiation and progression, neuron ablation emerged as a treatment strategy mentioned a lot. Some studies demonstrate that surgical or pharmacological denervation attenuate tumor growth in a tissue-specific manner. For instance, sensory neuron ablation is proved to favor melanoma progression, but slows the initiation and progression of pancreatic cancer (Saloman et al., 2016; Prazeres et al., 2020). And denervation suppresses gastric and breast tumorigenesis (Zhao et al., 2014). Although method to manoeuvre intra-tumoral innervations is not yet mature for clinical treatment, it provides a promising new avenue for anti-cancer therapy (Prazeres et al., 2020).

Apart from neuron ablation, antagonize β2-AR seems to be a more mature approach to block the neuroendocrine signal. Propranolol is a nonselective beta blocker used for cardiovascular indications for decades (Srinivasan, 2019). However, in recent years, propranolol has been applicated in the treatment of some kinds of tumors successfully, such as angiosarcoma (Wagner et al., 2018). Retrospective studies have observed a correlation between *β* blocker usage and increased overall survival among cancer patients (Nagaraja et al., 2013). Blockade of *β*-AR reduces tumor progression and upregulates the response to anti-CTLA4 therapy contributes to the formation of an immunosuppressive TME. In mouse fibrosarcoma models, propranolol increased the number of T cells, reduced the number of intra-tumoral MDSCs and altered the gene expression profile of tumor-associated macrophages significantly in the TME. Similar phenomenon was observed in murine models of colon cancer (Fjæstad et al., 2022). Specifically, one study demonstrates that NE-βAR pathway helps to maintain the level and the suppressive function of MDSCs. In murine breast cancer models, β2-adrenergic signaling modulated the expression of immunosuppressive molecules such as arginase-I and PD-L1 and suppressed the proliferation of T cells. Also, the author pointed out that the regulatory functions of β2-AR signaling in MDSCs is activated by STAT3 phosphorylation. The β2-AR-mediated increase in MDSC is dependent on Fas-FasL interactions. Besides, the immunosuppressive function of MDSCs can be decelerated by *β*-AR antagonists (Mohammadpour et al., 2019). Propranolol was also reported to reduce stress-induced elevation of regulatory T cells in breast cancer patients (Zhou et al., 2016). Another study report that β2-adrenergic signaling contributes to an exhausted phenotype in T cells and induce metabolic dysfunction in the TME. In mouse melanoma and colon cancer models, it is observed that by using propranolol, tumor growth rate was slowed accompanied by a significantly decrease of tumor-infiltrating T cells that express exhaustion related genes and an increase in progenitor exhausted T cells. Also, *β*-AR blockade in mice increases oxidative phosphorylation and glycolysis in tumor-infiltrating lymphocyte (Qiao et al., 2021). Therefore, *β*-AR antagonists such as propranolol could be a potentially efficacious therapy approach of tumors in the future.

In traditional Chinese medicine, moxibustion, a treatment modality with a long history, has been used to treat cancer-related symptoms in clinical practice for years. However, few people have explored its mechanism of action. Interestingly, a recent study informed that grain-sized moxibustion significantly reduced tumor growth in lung cancer. Grain-sized moxibustion performed at the acupoint of Zusanli promotes anti-tumor immunity of NK cell by inhibiting adrenergic signaling. The acupoint of Zusanli is an important acupoint involved in the interaction between neuroendocrine systems and immune cells (Zhang et al., 2018). *In vitro*, grain-sized moxibustion increased the proportion, infiltration and activation of NK cells, yet it didn’t affect T cells. Additionally, the grain-sized moxibustion mediated NK cells activation can be reduced by *β*-blocker treatment (Hu et al., 2021). To sum up, we describe the role of moxibustion in neuroendocrine regulation of lung cancer, which indicated that traditional Chinese medicine is still an ancient but promising therapeutic regimen for cancers.

## 5 Conclusion

Many studies have shown that tumor innervation is associated with tumor initiation, progression and a worse prognosis, and neuroendocrine regulation is the main mechanism by which nerves modulate tumor cells and other non-nerve cells. Several studies have focused on the neuroendocrine regulation of tumor cells; however, immune cells are also a vital part of the TME. Various tumor-associated immune cells have complex functions that can greatly impact tumor development, prognosis and treatment. Therefore, this review discusses the existing research on the neuroendocrine regulation of tissue-specific immunity. The regulatory mechanisms of different immune cell types in various tissues were summarized according to the classification of regulatory factors. Adrenergic and cholinergic signaling are two common regulatory pathways. In central and peripheral tissues, sympathetic nerves mainly modulate tissue-resident immune cells through the NE-β2A pathway, exerting anti-inflammatory and immunosuppressive roles. The parasympathetic nervous system regulates resident macrophages and ILCs mainly by secreting ACh from the vagus nerve to promote macrophage activity and anti-inflammatory effects. Other neurotransmitters are secreted primarily in the CNS and regulate microglia activity. Neuropeptides are another type of neuroendocrine factor that is secreted by central, autonomic and sensory neurons. These neuropeptides have various functions in tissues. Furthermore, many tissue-resident immune cells secrete cytokines and other factors to regulate nerves. We also summarized the available studies on neuroendocrine immunomodulatory regulation in different kinds of tumors. Some studies focused on autonomic nervous regulation in cancers and have helped to explain why chronic stress promotes tumor development from an immune aspect. Yet, many tumors are also regulated by sensory nerves and other factors. After that we discuss the neuroendocrine regulation of tumor immunity from the perspective of immune cell function and tumor therapy.

There are still knowledge gaps in the field of neuroendocrine regulation of immune cells in the TME. Many receptors of neuroendocrine factors are expressed on macrophages, T cells, and other cells that play important roles in tumor immunity. Some regulatory pathways have been studied in nontumor tissues, while their roles in tumors have not been studied. Moreover, a variety of cancers have been reported to be affected by tumor innervation, but the neuron-immune interactions in most cancers remain unclear. Thus, we also covered some articles that only discussed neuroendocrine regulation from the perspective of TME. Although they did not mention the neuroendocrine regulation of immune cells, we believe that some of them can provide ideas for the following research.

Due to the tissue specificity of immune cells, individualized immunotherapy in tumors may bring greater benefits to patients. For example, some studies have found that β2-AR blockade can inhibit NE-mediated tumor progression, but it has not been applied clinically in most kinds of tumors. Therefore, enriching the research focusing on these aspects may provide new insights for personalized tumor immunotherapy.

## Glossary

TME: tumor microenvironment
DC: dendritic cell
ILC: innate lymphoid cell
TRM: tissue-resident memory T cell
CNS: central nervous system
PNS: peripheral nervous system
NE: norepinephrine
AR: adrenergic receptor
NGF: nerve growth factor
TEM: effective memory T cell
ATM: adipose tissue macrophage
ASC: adipose stem cell
MM: muscularis macrophage
iNKT: invariant natural killer T
ACh: acetylcholine
mAChR: metabotropic muscarinic ACh receptor
nAChR: ionotropic nicotinic ACh receptor
5-HT: serotonin
GABA: gamma-aminobutyric acid
DA: dopamine
DR: dopamine receptor
5-HTR: 5-HT receptor
NMU: neuromedin U
SP: substance P
CGRP: calcitonin gene-related peptide
PACAP: pituitary adenylate cyclase-activating polypeptide
VIP: vasoactive intestinal peptide
NPFF: neuropeptide FF
NPY: neuropeptide Y
CRLR: receptor calcitonin receptor like receptor
PNEC: pulmonary neuroendocrine cell
MRGPR: Mas-related G-protein coupled receptor member
dLN: draining lymph node
ChAM: cholinergic adipose macrophage
BMP2: bone morphogenetic protein 2
CSF1: colony stimulatory factor
SAM: Sympathetic neuron-associated macrophage
NAM: nerve- and airway-associated macrophage
MDSC: myeloid-derived suppressor cell
DRG: dorsal root ganglia
PRP-1: proline rich polypeptide 1
SST: somatostatin
SSA: somatostatin analog
